# Skin, Heart, and CNS Involvement in Granulomatosis With Polyangiitis: A Case Report

**DOI:** 10.7759/cureus.65935

**Published:** 2024-08-01

**Authors:** Muhammad Bilal Mohsin, Uswah Rasool, Wissam A Saliba

**Affiliations:** 1 Internal Medicine, Shifa International Hospital Islamabad, Islamabad , PAK; 2 Nephrology, Ascension Via Christi St. Francis Hospital, Wichita, USA

**Keywords:** granulomatous-necrotic skin lesion, neurological deficit, mitral valve vegetation, valve vegetation, cns involvement, : acute kidney injury, granulomatosis with polyangiitis (gpa), anca associated vasculitis

## Abstract

We discuss the case of a 60-year-old male who presented with ankle pain, a necrotic rash, and progressive weakness in both lower limbs and the right upper limb. An infectious workup of the skin lesions came back negative. Additionally, his kidney function tests indicated an acute kidney injury. This prompted investigations for vasculitis etiologies, which revealed a positive cytoplasmic antineutrophil cytoplasmic autoantibody (c-ANCA). His neurological deficits were also investigated, and imaging suggested embolic infarcts. Cardiac imaging showed valve vegetations and blood culture showed a lack of growth suggestive of a noninfective nature of these lesions. Based on all these findings, a kidney biopsy was obtained and demonstrated pauci-immune segmental vasculitis consistent with ANCA-associated glomerulonephritis. As such, the patient showed improvement with heavy pulse steroid and immunomodulator therapy. Although skin, heart, and CNS involvement have been previously reported with ANCA-associated vasculitis, it is rare, especially together, and can prove a diagnostic challenge. Therefore, it is important to consider vasculitis etiology in patients presenting similarly. In addition, this case highlights the overlapping clinical picture between infective endocarditis and vasculitis with valvular involvement, making differentiation between the two challenging.

## Introduction

Antineutrophil cytoplasmic autoantibody (ANCA)-associated vasculitides (AAV) include a group of disorders that involve inflammation of small and medium-sized vessels. The autoimmune process is mediated by the presence of ANCA antibodies in the blood. This group of disorders has been further classified into three, based on clinical syndromes: granulomatosis with polyangiitis (GPA), microscopic polyangiitis (MP), and eosinophilic granulomatosis with polyangiitis (EGPA) [1]. 

Although any tissue can be involved in AAV, the respiratory tract and kidneys are most commonly and severely affected [2]. GPA typically presents as chronic sinusitis, arthralgias, lung nodules, and acute kidney injury alongside constitutional symptoms. Leukocytoclastic skin involvement may also be seen. This clinical syndrome is accompanied by PR3-ANCA positivity [3].

This case study is noted to have atypical systemic involvement of the CNS, heart, and skin alongside the kidneys. 

## Case presentation

**Figure 1:**
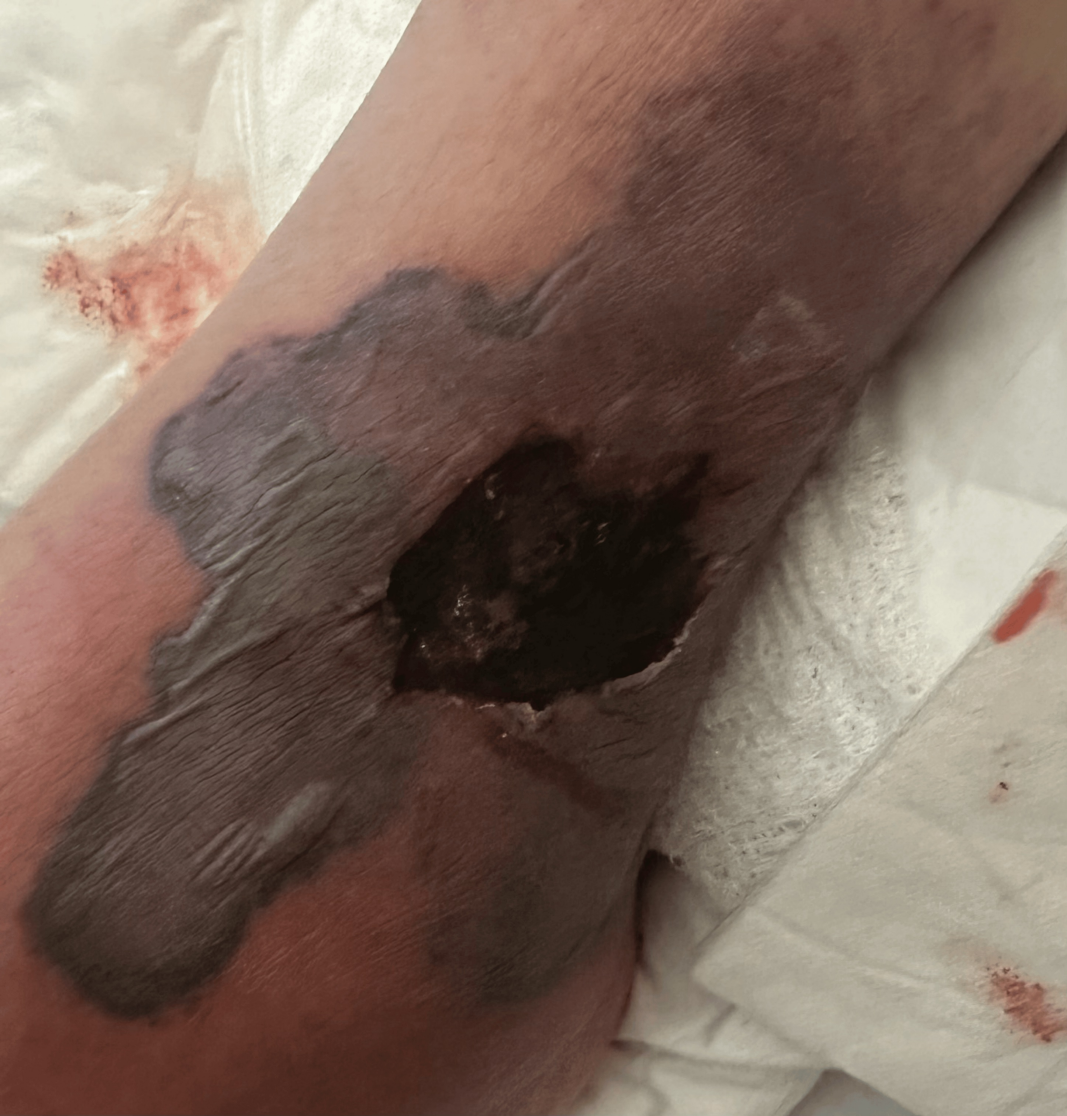
Necrotic lesion on the lateral aspect of the left ankle.

**Figure 2:**
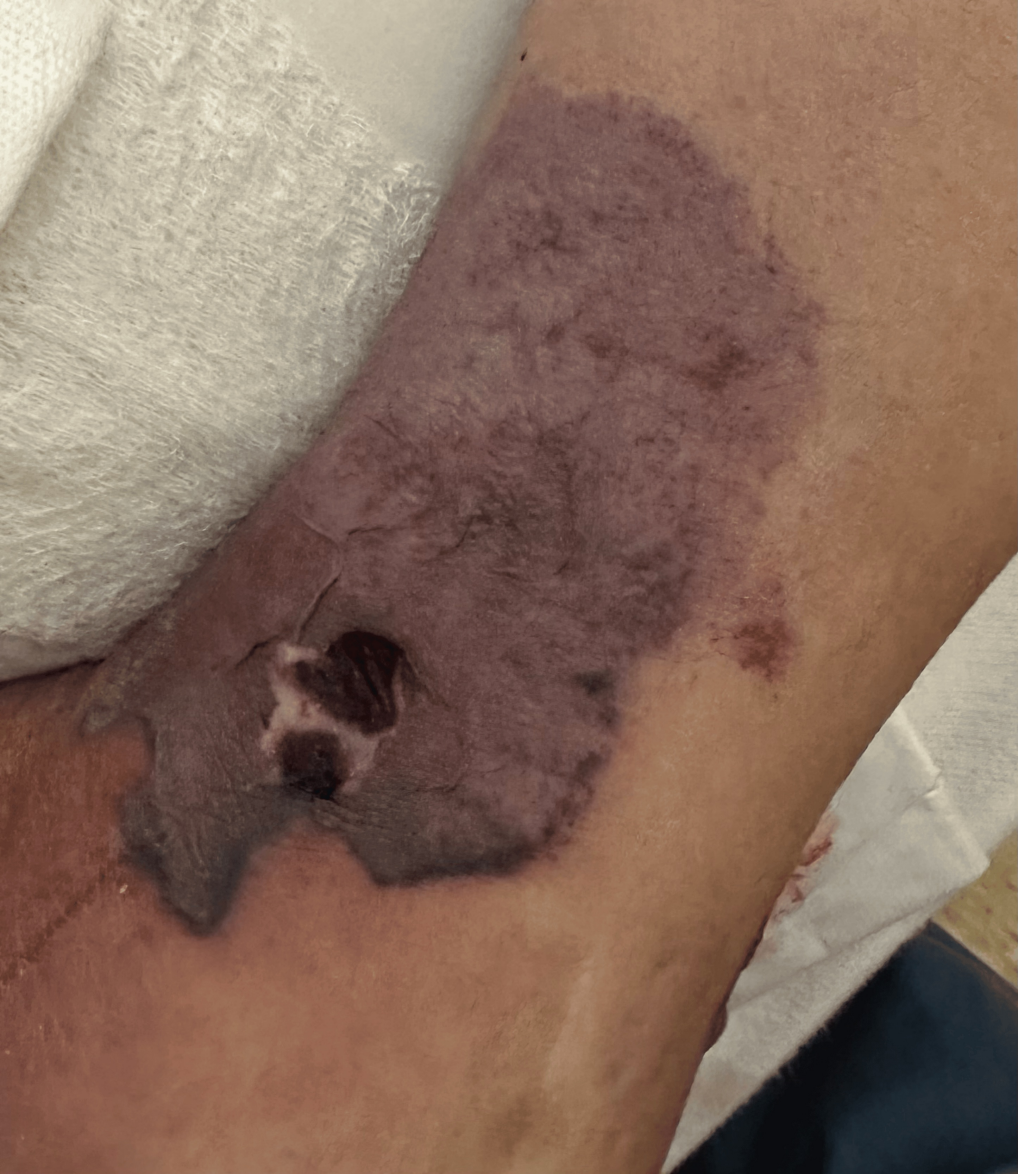
Necrotic lesion on the medial aspect of the left ankle.

**Figure 3:**
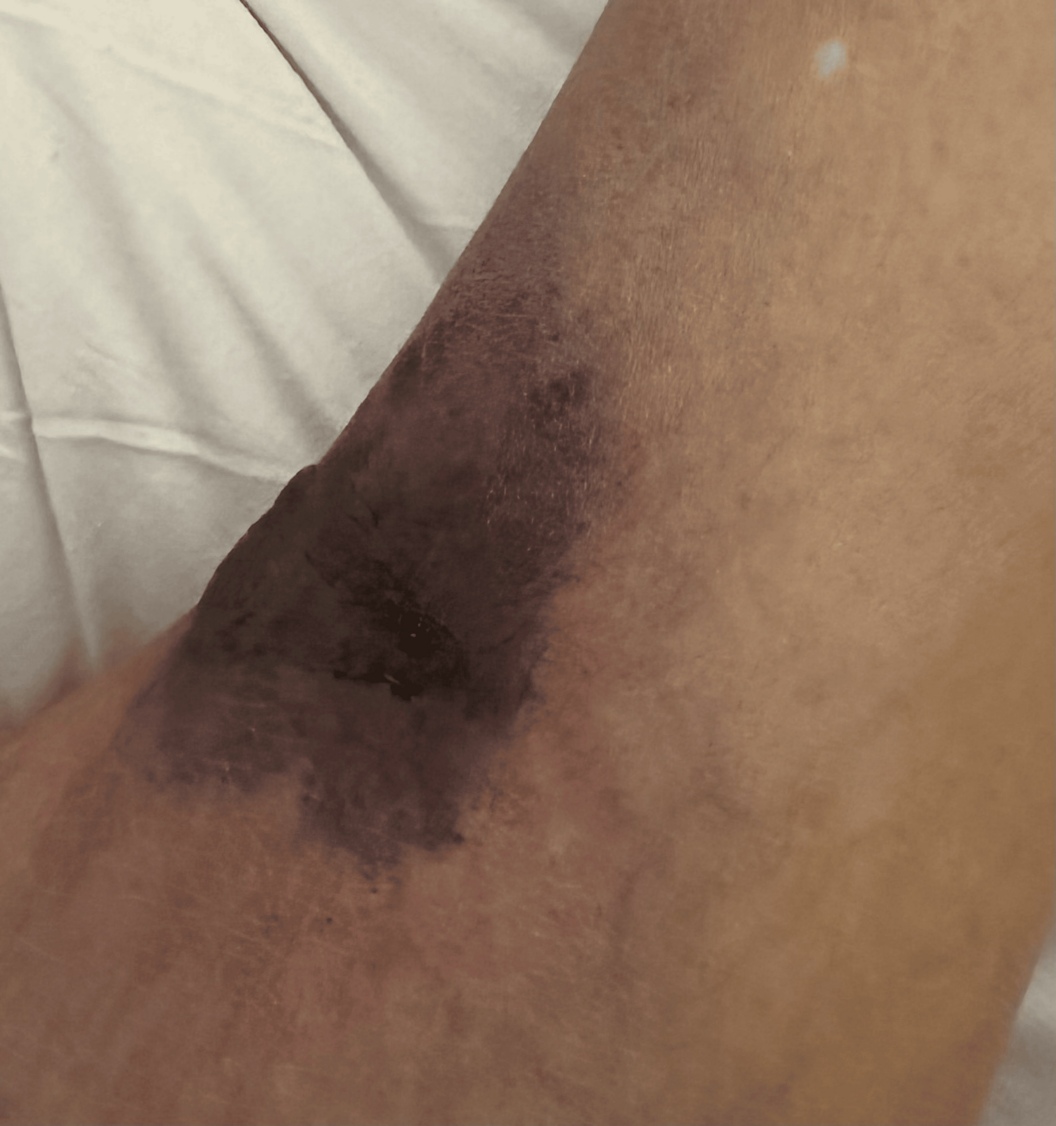
Necrotic lesion on the lateral aspect of the right ankle.

**Figure 4:**
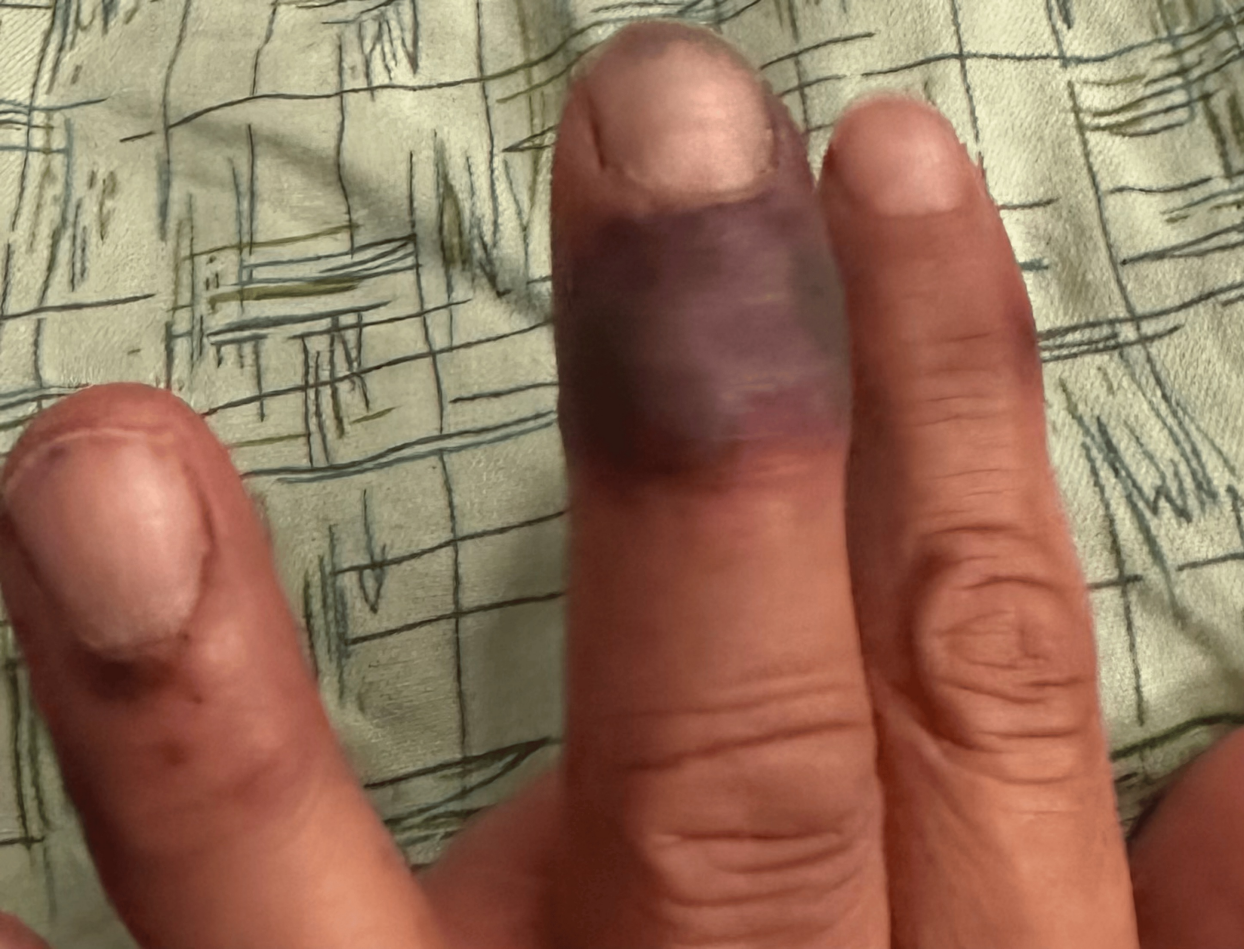
Lesions can be seen on the patient's left hand, most notably involving the middle finger.

A 60-year-old male presented with pain, swelling, and a rash on both ankles that had been present for three weeks. The patient could not bear weight, and the rash began to blister and ooze. He had some edema and necrotic ulcers with a surrounding black plaque on the left ankle (Figures 1-2) and a similar, smaller hemorrhagic lesion on the right ankle (Figure 3). The rash had also progressively involved his fingers (Figure 4). He was initially diagnosed with cellulitis and given empiric antibiotics. A non-contrast CT of the left ankle showed soft-tissue swelling with degenerative changes. Ultrasound Doppler of the lower extremities showed no evidence of deep venous thrombosis bilaterally. Detailed examination of the lesions gave rise to the suspicion of a noninfectious etiology. A negative Gram stain and culture confirmed confirmed this. 

His initial laboratory results (Table 1) and urinalysis findings (Table 2) are presented below.

**Table 1:** Patient labs at the time of admission. WBC, white blood cells; Hb, hemoglobin; ESR, erythrocyte sedimentation rate; CRP, c-reactive protein; INR, international normalized ratio; Na, sodium; K, potassium; HCO_3_, bicarbonate; BUN, blood urea nitrogen; Cr, creatinine; eGFR, estimated glomerular filtration rate; Ca, calcium

Labs	Value	Normal range	Unit
WBC	26.4	4.5-11.0	x10^9 ^/L
Hb	11.9	13.0-17.0	g/dL
Platelets	307	150-400	x10^9^/L
ESR	38	<20	mm/hour
CRP	31.4	<0.3	mg/dL
INR	1.4	<1.1	-
D-dimer	3,163	<500	ng/mL
Na	133	135-145	mEq/L
K	3.7	3.5-5.2	mEq/L
HCO_3_	17	22-28	mEq/L
BUN	71	6-24	mg/dL
Cr	2.5	0.7-1.3	mg/dL
eGFR	26	>90	mL/min/1.73 m^2^
Ca	8.2	8.5-10.2	mg/dL
Albumin	2.2	3.5-5.5	g/dL

**Table 2:** Abnormal urinalysis findings. The presence of 3+ blood indicates significant microscopic hematuria. This was supplemented with the visualization of red blood cells. The 1+ protein indicates slight proteinuria corresponding to approximately 30 mg of protein per dL of urine.

Parameter	Result	Reference
Blood	+++	Negative
Protein	+	Negative

The elevated BUN and Cr and decreased GFR alongside his urinalysis report indicated an acute kidney injury. These findings, in addition to an elevated ESR, and the ankle lesions, strengthened the suspicion of a primary vasculitis. A renal ultrasound was unremarkable. He underwent an autoimmune workup with the findings given in Table 3.

**Table 3:** Patient's immunological lab findings. c-ANCA, cytoplasmic antineutrophil cytoplasmic autoantibody; ANA, antinuclear antigen

Labs	Value	Normal range	Unit
c-ANCA	2149	<2.8	units/mL
C4 complement	15	15-45	mg/dL
C3 complement	98	88-201	mg/dL
ANA	Negative	Negative	-

Core needle biopsies of the right kidney demonstrated pauci-immune segmental vasculitis consistent with ANCA-associated glomerulonephritis. Immunofluorescence of the specimen depicted focal mesangial staining for IgA (2+), IgG (focal and segmental 2+), kappa (2+), and lambda (2+) along with mesangial entrapment for IgM (1+) and C3 (2+). 

During his hospitalization, he developed asymmetric bilateral lower extremity weakness which progressed to involve his right upper limb as well. On physical examination, he demonstrated a complete inability to move his toes prompting a non-contrast CT scan of the head to be ordered, however, this was unremarkable. This was followed by a non-contrast MRI of the head which revealed multifocal areas of diffusion restriction seen in the periventricular white matter, basal ganglia, right cerebellar hemisphere, and posterior right corpus callosum, suggestive of acute-subacute embolic ischemic infarcts (Video 1). Ultrasound of the carotids showed the bilateral carotid bifurcations to be patent. Subsequent transesophageal echocardiography revealed a normal ejection fraction, moderate left ventricular hypertrophy, moderate to severe mitral insufficiency, and prolapse of the posterior leaflet along with findings suggestive of vegetations on the mitral valve. The blood cultures came back negative, indicating that these vegetations were noninfectious. 

**Video 1:** The MRI of the patient's brain.

The antibiotics were discontinued, and the patient was started on Solu-Medrol 1,000 mg intravenously (IV) daily for three days, followed by oral prednisone and four doses of rituximab. He required a short course of dialysis to establish a baseline creatinine of 1.4. On follow-up, his prednisone dose was tapered down to 10 mg before being discontinued, and he was maintained on avacopan 30 mg daily, achieving disease remission.

## Discussion

The 2022 American College of Rheumatology classification criteria for GPA include clinical, lab, and imaging parameters for diagnosis of GPA. Clinically, it included nasopharyngeal symptoms, cartilaginous involvement, hearing loss, and lung involvement. Imaging and lab criteria included upper or lower respiratory tract lesions on imaging, PR3-ANCA positivity, pauci-immune glomerulonephritis, and granulomatous inflammation on biopsy. The criteria have been validated for use in research [4]. Any organ may be involved in a systemic inflammatory process, however, this criterion supports the understanding that the nose, lungs, and kidneys are the most clinically relevant organs affected by GPA. Although skin involvement may be frequently noted in AAV, it is not typically a common presentation for GPA. 

Cutaneous lesions have been noted in all subtypes of AAV. Thirty-four percent of patients with GPA were found to have skin involvement [5]. The most frequently seen manifestations were petechiae or purpura. As highlighted in the case, our patient had skin lesions at the time of onset of symptoms. Erythematous papules associated with a flare of GPA have been reported in a case previously, which progressed to hemorrhagic blisters. Histopathological evaluation revealed interstitial granulomatous dermatitis and foci of dermal hemorrhage [6]. Our patient did not undergo histological evaluation; however, it can be extrapolated from the gross inspection of the lesions and the infectious workup being negative that the cutaneous lesions in our patient were secondary to underlying vasculitis. Skin lesions, although an established association with AAV do not usually present with necrotic ulcers to the extent seen in our patient. The active vasculitis initially causes blistering and the progression to necrotic ulceration may be a gross depiction of the underlying progression of inflammation to ischemic vasculopathy. This has been seen in a previously reported case as leukocytoclastic vasculitis with fibrinoid necrosis of the vessel walls [6]. 

Neurologic involvement is seen in <15% of the patients with AAV. Of these entities, neurologic involvement is most frequently seen in patients with GPA, ranging from 22-54%. CNS can be affected by the inflammation of vessels present in the CNS tissue, infiltration of granulomatous pathology from nearby structures, or by granulomatous inflammation of the CNS tissue itself. Symptoms usually present late in the disease course [7]. Our patient presented initially with asymmetric bilateral weakness of the lower limbs progressing to involve the right upper limb. These neurological deficits were not explained entirely by the findings of the MRI scan suggesting that multiple pathogenesis may be at play. 

Ischemic infarctions and intracranial hemorrhages, although rare, have been seen at the time of presentation in AAV. These typically present as isolated or multiple lesions affecting the white matter. Distal penetrating vessels are most frequently affected by vasculitis. Imaging findings may show ischemic or hemorrhagic lesions affecting white and gray matter. Nonspecific white matter lesions can appear in periventricular and subcortical regions including the basal ganglia, the midbrain, and the pons. This is similar to what we found on brain MRI in our patient. It was suggested that the lesions appeared to be embolic, which led to further workup. We found noninfectious vegetation on the mitral valve, which could have been the likely source of emboli. This presentation is atypical for GPA as per the previously reported cases. On the other hand, the ischemic infarcts seen may as well have been non-embolic, purely secondary to inflammatory changes in the vasculature. 

Despite cardiac involvement being recognized as an increasingly frequent aspect of AAV, with 6% to 44% of cases of GPA showing cardiac signs and symptoms [8], these are still a more infrequent occurrence in comparison to the involvement of the nose (92%), lung (85%), and kidneys (77%). The most common of these are noted to be pericarditis, myocarditis, coronary arteritis, aortic valve regurgitation, and conduction system defects [9,10] across various studies, with mitral valve regurgitation and valvular vegetations being seen much more rarely [8,11]. As such, it may become especially difficult to differentiate infective endocarditis from GPA, as in the case of our patient, with initial manifestations being very similar in both conditions, including cutaneous, renal, pulmonary, and neurological lesions [12,13]. Furthermore, elevated acute-phase reactants and transient infection-induced increases in ANCA levels can complicate the clinical picture [12]. Therefore, it is essential to consider the possibility of GPA in patients with cardiac involvement, especially when blood cultures are negative and ANCA positivity is strong.

Our patient’s response to treatment with glucocorticoid, rituximab, and avacopan is confirmatory of the diagnosis of GPA despite the unusual presentation. 

## Conclusions

We describe an atypical case of GPA, highlighting the rare simultaneous involvement of the CNS, heart, and skin, in addition to the usual involvement of the kidneys, which presents a complex clinical picture. The patient initially presented due to progressive bilateral ankle lesions and associated pain. Extensive workup on the patient revealed multi-systemic involvement. A renal biopsy and elevated c-ANCA levels confirmed the diagnosis of GPA. The patient was started on a 1,000 mg IV Solu-Medrol pulse dose for three days followed by oral prednisone and four doses of rituximab. The prednisone was tapered and discontinued, and disease remission was maintained with avacopan 30 mg daily. With this report, we aim to highlight an important differential diagnosis to consider, even in cases that deviate from the typical presentations of GPA.
